# The Role of Retinal Carotenoids and Age on Neuroelectric Indices of Attentional Control among Early to Middle-Aged Adults

**DOI:** 10.3389/fnagi.2017.00183

**Published:** 2017-06-09

**Authors:** Anne M. Walk, Caitlyn G. Edwards, Nicholas W. Baumgartner, Morgan R. Chojnacki, Alicia R. Covello, Ginger E. Reeser, Billy R. Hammond, Lisa M. Renzi-Hammond, Naiman A. Khan

**Affiliations:** ^1^Department of Kinesiology and Community Health, University of Illinois at Urbana–ChampaignUrbana, IL, United States; ^2^Division of Nutritional Sciences, University of Illinois at Urbana–ChampaignUrbana, IL, United States; ^3^Department of Psychology, University of GeorgiaAthens, GA, United States; ^4^Neuroscience Program, University of Illinois at Urbana–ChampaignUrbana, IL, United States

**Keywords:** cognitive aging, macular pigment optical density, lutein, carotenoids, event-related potentials, inhibition, cognitive control

## Abstract

One apparent consequence of aging appears to be loss of some aspects of cognitive control. This loss is measurable as early as mid-adulthood. Since, like many aspects of cognition, there is wide variance among individuals, it is possible that behavior, such as one’s diet, could drive some of these differences. For instance, past data on older humans and non-human primates have suggested that dietary carotenoids could slow cognitive decline. In this study, we tested how early such protection might manifest by examining a sample (*n* = 60) of 25–45 year olds. Carotenoid status was assessed by directly measuring macular pigment optical density (MPOD) which has shown to be highly correlated with the primary carotenoid in brain, lutein. Cognitive control was measured using event-related potentials during the performance of cognitive control tasks designed to tap into different aspects of attentional (i.e., selective attention, attentional inhibition, and response inhibition) control. Our results showed that, across participants, MPOD was related to both age and the P3 component of participants’ neuroelectric profile (P3 amplitude) for attentional, but not response, inhibition. Although younger adults exhibited larger P3 amplitudes than their older adult counterparts, older subjects with higher MPOD levels displayed P3 indices similar to their younger adult counterparts in amplitude. Furthermore, hierarchical regression analyses showed that age was no longer a significant predictor of P3 amplitude when MPOD was included as a predictor in the model, suggesting that MPOD may partially contribute to the relationship between age and P3 amplitude. In addition, age and MPOD were shown to have independent associations with intraindividual variability of attentional control, such that younger individuals and individuals with higher MPOD showed less intraindividual variability. These results show a relationship between retinal carotenoids and neuroelectric indices underlying cognitive control. The protective role of carotenoids within the CNS may be evident during early and middle adulthood, decades prior to the onset of older age.

## Introduction

Lutein (L) and zeaxanthin (Z) are naturally occurring carotenoids found in abundance in richly colored fruits and vegetables (e.g., spinach and kale). These pigments cannot be synthesized de novo, and thus must be obtained from the diet. Since they can also be directly and non-invasively measured in central nervous system tissue (the retina), and those levels correlate strongly with dietary intake, serum levels, and brain concentrations (Burke et al., 2005; Vishwanathan et al., 2015), they provide a powerful biomarker and means of testing how diet might influence the brain. Lutein is the predominant carotenoid in human brain tissue and greater lutein status is positively associated with better cognitive function in older adults (Johnson et al., 2008; Feeney et al., 2013; Johnson et al., 2013; Renzi et al., 2014; Vishwanathan et al., 2014a). Whether greater accumulation of retinal carotenoids is related to cognitive abilities and underlying neuroelectric function in earlier adulthood, however, has not been as thoroughly studied.

Much effort has been directed toward determining the cognitive constructs that are affected in normal aging and the underlying mechanisms that may explain age-related cognitive decline. Considerable research has shown age-related decline across a variety of cognitive domains including attention (Cabeza et al., 2004), working memory (Kirova et al., 2015), inhibition (Sebastian et al., 2013; Pettigrew and Martin, 2014), context processing (Rush et al., 2006), executive functioning (Silver et al., 2011), and processing speed (Albinet et al., 2012). Furthermore, brain-based research has begun to elucidate the mechanisms by which cognitive aging occurs. For example, fMRI work has shown that older adults tend to exhibit more widespread brain activation than younger adults during some cognitive tasks (Cabeza, 2002; Payer et al., 2006; Schneider-garces et al., 2009), suggesting the need for a greater recruitment of neural resources for successful task completion. In addition, older adults show less correlation of activation among different brain regions, suggesting less integrated functioning across the brain (Andrews-Hanna et al., 2007).

Traditionally the work on cognitive aging has been done cross-sectionally by comparing older adults with either middle aged or younger adults. There is evidence to suggest, however, that cognitive aging has its origins much earlier than older adulthood (Salthouse, 2005, 2009). For example, work done in samples of 18–84 year olds shows a significant relationship between age and a host of cognitive outcomes across the adult lifespan (Salthouse, 2001; Salthouse et al., 2003). Decreased performance in middle age may be especially prevalent when cognitive load is increased (Jain and Kar, 2014), and especially among low performers (Vuoksimaa et al., 2013). Imaging studies have identified neural patterns that mimic the kind of loss one typically sees in the elderly in non-clinical populations of early and middle aged adults including decreased grey matter volume (Sowell et al., 2003; Allen et al., 2005), whole brain volume (Fotenos et al., 2005), regional white matter volume (Pieperhoff et al., 2008), and cortical thickness (Magnotta et al., 1999; Salat et al., 2004).

Event related potential (ERP) work has shown consistent age-related changes in the P3 component across the lifespan. Early work established that age-related changes can be seen in P3 latency, with latencies decreasing over the course of adulthood, reaching a peak in early adulthood, and subsequently increasing over the course of middle to late adulthood (Courchesne, 1978; Goodin et al., 1978; Beck et al., 1980; Brown et al., 1983). Later work, however, has shown a similar trend in P3 amplitudes, with amplitudes increasing throughout childhood and decreasing beginning in middle age. Mullis et al. (1985), for example, showed that in a sample of 8–90 year old participants completing a modified oddball task, participants showed increasing P3 amplitudes to target stimuli up to 30–35 years of age, after which amplitudes decreased through older age. More recent work has shown greater differentiation in P3 amplitudes to target and standard stimuli across the lifespan and found decreased differentiation as a function of age beginning in middle age and increasing into older adulthood. This effect was driven by an age-related trend of larger amplitudes elicited for standard stimuli and smaller amplitudes for targets across middle and older age (Mott et al., 2014). Furthermore, it has been shown that the scalp distribution of the P3 changes across the lifespan, with healthy younger adults showing a posterior maxima which becomes increasingly central and more widespread with age (Fabiani and Friedman, 1995; Friedman et al., 1998; Lorenzo-López et al., 2007; Vallesi, 2011). The N2, a component thought to be a marker of inhibition (Jodo and Kayama, 1992), is shown to peak in amplitude and latency during childhood and subsequently decrease in adolescence through early adulthood (Johnstone et al., 2005). A continued slowing of the N2 response has been shown in older adulthood for visual stimuli (Falkenstein et al., 2002).

If, in fact, cognitive loss can begin so early, it is reasonable to question whether such changes can be delayed or prevented through changes in one’s lifestyle. Much of this work has focused on the role of physical fitness and activity, indicating that older adults who are physically active or have superior cardiovascular health show fewer age-related declines (van Boxtel et al., 1997; Pontifex et al., 2009; Wendell et al., 2014; Bula, 2016), and that exercise interventions may serve to protect against cognitive aging (Colcombe and Kramer, 2003). These effects have been shown across the lifespan (van Boxtel et al., 1997; Hillman et al., 2006). Work on dietary interventions (Johnson et al., 2008; Tucker, 2016) is more recent. The majority of work in this field has targeted specific nutrient supplementation, with evidence suggesting that vitamin D (Hooshmand et al., 2014), vitamin B (Erickson et al., 2008; Clarke et al., 2014), and omega 3 fatty acids (Conklin et al., 2007; Dangour et al., 2012; Konagai et al., 2013) act as neuro-protective agents in aging adults. Carotenoids have also been studied with most attention being focused on L and Z. These antioxidant pigments have had a long history of being studied for their role in ocular function and disease (e.g., Stringham et al., 2008; Tan et al., 2008; Hammond et al., 2012), as well as a multitude of systemic health issues such as cancer, cardiovascular disease, and neurodegenerative disease (Krinsky and Johnson, 2005).

Macular pigment optical density (MPOD), a cumulative biophysical measure of carotenoids known to accumulate in the retina (i.e., L, Z, and mesozeaxanthin), has been specifically linked to cognitive and brain health (Johnson, 2014). As such, greater MPOD has been correlated with superior visual processing abilities (Renzi and Hammond, 2010) and decreased risk of macular degeneration (Snodderly, 1995; Tan et al., 2008). However, in addition to accumulation in the macula, L has been shown across brain cortices in both infants and older adults (Craft and Dorey, 2004; Vishwanathan et al., 2014b; Erdman et al., 2015; Lieblein-boff et al., 2015). L and Z have anti-oxidative and anti-inflammatory properties (Beatty et al., 2000; Pintea et al., 2011) but also likely influence brain function through a number of other possible mechanisms (reviewed by Johnson, 2014). For example, Vishwanathan et al. (2014a) tested older adults in a battery of cognitive tasks and found that their retinal L and Z levels significantly corresponded to global cognitive abilities including verbal learning, verbal fluency, memory recall, processing speed, and perceptual speed. L and Z levels have also been shown to protect cognitive function in older adults with mild cognitive impairment (Renzi et al., 2014) and supplements of L, Z and DHA have shown to improve verbal fluency and memory in healthy older women (Johnson et al., 2008). However, the extent to which retinal carotenoids may interact with age on specific aspects of cognitive function remains inadequately studied, particularly among young or middle-aged adults.

The aims of the present study were to: (1) establish any independent relationships between age, retinal carotenoid levels (MPOD), and ERP indices in early and middle-aged adults (25–45 years); and (2) investigate the nature of the relationship between age, MPOD, and neuro-cognitive indices during tasks eliciting selective attention, attentional inhibition, and response inhibition. We hypothesized that age would be negatively associated with MPOD and positively associated with behavioral performance in both tasks, as well as to the corresponding ERP indices. Further, we hypothesized that both age and MPOD would be related to neuro-cognitive indices, but that the adjusting of MPOD would significantly influence the contribution of age.

## Materials and Methods

### Participants

Cross-sectional data were collected from 60 adult participants between the ages of 25–45 years old living in the Eastern-Central Illinois region. To qualify for the study, participants had to provide all demographic data, complete the Kaufman Brief Intelligence Test (KBIT), a measure of intelligence quotient (IQ) (Kaufman and Kaufman, 1990), provide a readable EEG recording, have normal or corrected to normal vision based on the minimal 20/20 standard, be free of diagnosed neurological disorders and diseases (e.g., ADD/ADHD and autism). Participants were excluded if they did not complete all relevant aspects of testing, if they were not in the selected age range, if they were pregnant or nursing, or if they were currently taking any anti-psychotic, anti-depressant, or anti-anxiety mediations. All participants provided verbal and written consent in accordance with the University of Illinois’ Institutional Review Board and the Declaration of Helsinki.

### MPOD Assessment

Macular pigment optical density was measured with a customized hetero-flicker photometry (cHFP) technique and a macular densitometer (Macular Metrics Corporation, Rehoboth, MA, United States). This technique and its underlying principles have been described previously (Wooten et al., 1999; Hammond et al., 2005). In short, participants are asked to view stimuli peaking at a measuring wavelength of 460 nm that flickers in counterphase with a 570 nm reference (flicker rate being optimized for the optimal width of the subject’s null zone). Participants were asked to adjust the radiance to identify a null flicker zone by indicating when they could no longer detect the flicker. The task is done while the stimulus is centrally fixated (measuring MP where it is most dense) and at 7° in the para-fovea (where density is minimal). The MPOD is calculated by subtracting the foveal from the parafoveal log sensitivity measurements after normalizing at 570 nm.

### Cognitive Tasks

A two-stimulus oddball task was used to assess selective attention. In this task, participants viewed a series of large and small circles presented serially. Large circles (5.5 cm diameter) served as the “rare” stimulus and were presented on 20% of trials in a random order, whereas the “frequent” small circle stimuli (3 cm diameter) were presented in the remaining 80% of trials. Participants were to respond to the rare target trials with a button press. Participants were presented with a practice block of 30 trials followed by 200 experimental trials. Stimuli were presented for 100 ms with a 1000 ms response window and an inter-trial interval of 2000 ms. This task is described in detail in Pontifex et al. (2009).

The Eriksen flanker task was used to assess attentional inhibition. In this task, participants viewed a sequential array of visually presented arrows. The arrows were white line drawings presented on a black background for 83ms. Participants were asked to attend to a centrally located target arrow presented amid an array of four task-irrelevant distractor arrows that flank the target on both sides. The task was to quickly and accurately respond to the directionality of the centrally located target arrow with a button press. The target was presented as either a congruent trial in which the flanking arrows face in the same direction as the target (>>>>>), or as an incongruent trial in which the flanking arrows face in the opposite direction in relation to the target (>><>>). After receiving instructions, participants completed a block of 40 practice trials. Following the practice trials, participants completed 200 experimental trials (2 blocks of 100 trials each), made up of an equiprobable distribution of congruent and incongruent trials as well as left and right target trials presented in a random order. The trials were presented with randomly selected inter-trial intervals of 1100, 1300, and 1500 ms.

A go/no-go task was used to assess response inhibition. In this task, participants were presented with the same large and small stimuli that were used in the oddball task (described above). However, in this task, participants are presented with a “go” stimulus (the 3 cm circle) in which they are to respond with a button press, as well as randomly interspersed “no-go” stimulus (the 5 cm circle) that they must ignore by inhibiting the established pre-potent, button-pressing response. As in the oddball task, participants were presented with a practice block of 30 trials followed by 200 experimental trials, and stimuli were presented for 100 ms with 1000 ms response windows and inter-trial intervals of 2000 ms. It should be noted that the go/no-go task was always presented directly after the oddball task, creating an even stronger pre-potent response to the rare (no-go) stimuli.

### ERP Recording Technique

Electro-encephalographic (EEG) activity was recorded via a Neuro-scan Quik-cap with 64 scalp electrodes arranged in the international 10–10 system. A midline sensor placed between Cz and CPz served as a reference and AFz served as the ground. Using a Neuroscan Synamps2 amplifier, continuous EEG signal was digitized at a sampling rate of 500 Hz, amplified 500 times to 70-Hz filter with a direct current and a 60-Hz notch filter. Electro-oculographic (EOG) activity was recorded with a set of four electrodes placed at the outer canthus of each eye and above and below the left orbit.

Offline, continuous data were re-referenced to the average mastoids. An independent components analysis (ICA) was used to systematically reject eye-blink artifacts from the data. Data were submitted to a 0.1 Hz high-pass filter before being submitted to the ICA. If a component identified during the ICA were correlated at or above 0.35 with the vertical EOG channel, it was considered an eye-blink and subsequently rejected. –200 to 1200 ms around stimulus onset was used as a time window for creating stimulus-locked epochs with a –200 to stimulus onset used for baseline correction. A 30-Hz zero phase shift low-pass filter was employed. Only trials that were responded to correctly were included in ERP analysis. Epochs were excluded if the moving window peak-to-peak amplitude exceeded ±100 100 μV using a 100 ms window and a 50 ms window step. ERP variables of interest were the P3 peak amplitude and peak latency. In the flanker task, the P3 was defined as the localized peak and corresponding latency occurring between 300 and 600 ms after stimulus onset; in the Oddball and Go/No-go tasks, it was defined as the localized peak and corresponding latency occurring between 350 and 750 ms after stimulus onset. The N2 in the go/no-go task was defined as the localized negative peak and corresponding latency occurring between 200 and 350 ms after stimulus onset.

### Statistical Analysis

All analyses were conducted using SPSS version 24 (IBM). Bivariate correlations were used to assess the relationships between the demographic variables of interest, MPOD, the cognitive variables, and the ERP variables of interest. To assess the relationships between the demographic variables of interest, MPOD, and the cognitive and ERP variables, three sets of bivariate correlations were conducted, the first using cognitive and ERP data from the oddball task, the second from the flanker task, and the third from the go-nogo task. The demographic variables of interest submitted to correlational analyses were participant age, sex, KBIT score, and family income. The cognitive variables of interest in the flanker task were mean reaction time, response accuracy, inverse efficiency (reaction time/accuracy), and coefficient of variation (CV) (SD reaction time/M reaction time). Inverse efficiency is a way to examine how quickly participants respond accounting for accuracy. Higher numeric values for inverse efficiency indicate less efficient response patterns. CV is a means of examining the degree of intraindividual variability or consistency in response times for correct trials. Previous work indicates that measures of dispersion such as intraindividual variability may be more sensitive to cognitive dysfunction than measures of central tendency (Leth-Steensen et al., 2000; Hervey et al., 2006; MacDonald et al., 2006). Higher CV values indicate less consistent response patterns and represent a hallmark of several conditions in which attention regulation is diminished including old-age and neurological disorders (Kelly et al., 2008). For both the oddball and the go/no-go tasks, mean reaction times, response accuracies, and inverse efficiency scores were used. For all performance indices, the accuracy is recorded as the percentage correct, and reaction time is based on the mean of trials that were responded to correctly in milliseconds. The ERP variables of interest for all tasks were the peak amplitude and peak latency at the PZ electrode, where P3 is maximal in typical healthy adults (Polich, 2007). For the go/no-go task, N2 is also reported at the FCZ electrode, as it is considered to index inhibitory control in this task (Jodo and Kayama, 1992). Two-tailed tests are reported, with alpha of.05 for determining statistical significance. Outliers were calculated and subsequently removed based on the dependent variables of interest. The outlier labeling rule (Hoaglin and Iglewicz, 1987), which creates high and low cutoff scores based on the overall distribution of data, was used to identify outliers.

In order to draw more specific conclusions regarding the role of age and MPOD in predicting the neuro-cognitive outcomes, a series of hierarchical linear regression (HLR) analyses were conducted. The variables included in these models were derived from the bivariate correlations. Of particular interest were variables significantly associated with both age and MPOD. In the flanker task, two neuro-cognitive outcomes were associated with age and MPOD: The peak amplitude of the incongruent flanker trials at the PZ electrode, and the CV for the incongruent flanker trials. Subsequently, HLR models were run with each of these variables as the dependent measure. In each case, age was entered as a predictor into block 1 of the model, and MPOD was entered as a predictor into block 2 of the model. Standardized betas with corresponding t values are reported for predictors and F values with R^2^ changes are reported for overall model fit. For the go-nogo task, no variables were significantly correlated with both age and MPOD scores; therefore, no subsequent HLR analyses were conducted.

## Results

Participants spanned early to middle adulthood, ranging in age from 25 to 45 years (*M* = 33.8, *SD* = 5.7, 31 females) and were normally distributed in terms of their KBIT scores (*M* = 110.2, *SD* = 13.3) and MPOD (*M* = 0.49, *SD* = 0.25) values (Shapiro Wilk values = 0.986,0.983, *p* = 0.711,0.554, respectively). Age and income, however, were not normally distributed, with our sample representing a disproportionately younger demographic. The distribution of family income was even, suggesting that our sample equally represented adults of various income classes (*N* ≤ $50,000 = 34, *N* > $50,000 = 22).

### Oddball Task

**FIGURE 1:**
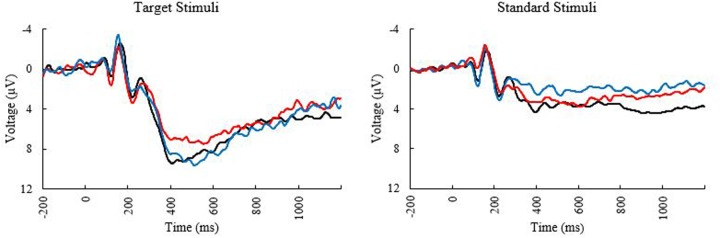
Event related potential (ERP) Waveforms at electrode PZ elicited during the oddball task. Black line, younger adults; blue line, older adults with low MPOD; red line, older adults with high MPOD.

Means and standard deviations of performance on the oddball task are shown in **Table 1**; results of the bivariate correlations are shown in **Table 2**. Waveforms, separated across the median values for age and MPOD, can be seen in **Figure 1**. The results indicate that participants performed well on the task overall, and while P3 amplitudes were numerically higher and latencies numerically lower on the targets compared to standards, as expected, neither were correlated with any of the demographic variables of interest. However, age was inversely correlated with response accuracy to standard stimuli (*r* = –0.266, *p* = 0.040), suggesting that younger adults had higher scores when asked to withhold responses to frequent, standard stimuli. MPOD was positively associated with both reaction time (*r* = 0.366, *p* = 0.004) and inverse efficiency (*r* = 0.257, *p* = 0.047) to target stimuli, suggesting that those with higher MPOD scores likely adopted a response set strategy in which they responded more slowly and less efficiently than their peers with lower MPOD. Finally, KBIT was positively associated with both the response accuracy to standard stimuli (*r* = 0.342, *p* = 0.007) and with reaction time to target stimuli (*r* = 0.468, *p* ≤ 0.001).

**Table 1:** Behavioral performance and event related potential (ERP) peak indices for the PZ electrode in the oddball task.

	Targets	Standards
Response accuracy (% correct)	84.6 (15.4)	90.4 (15.7)
Reaction time (ms)	477.9 (67.9)	-
Inverse efficiency	6.0 (2.0)	-
Peak amplitude (μV)	13.3 (8.6)	7.4 (9.2)
Peak latency (ms)	543.4 (124.9)	521.5 (131.8)


*Means are presented with standard deviations in parentheses.*

**Table 2:** Bivariate correlations between participant demographic characteristics, MPOD, and the neuro-cognitive data from the oddball task.

	Age	MPOD	Sex	KBIT	Income
**Accuracy**					
Targets	0.184	-0.167	-0.093	-0.095	0.085
Standards	-0.266*	0.162	-0.094	0.342**	0.147
**Reaction Time**					
Targets	-0.195	0.366*	0.014	0.468**	0.005
Standards	-	-	-	-	-
**Inverse Efficiency**					
Targets	-0.094	0.257*	-0.014	0.222	0.051
Standards	-	-	-	-	-
**Peak Amplitude**					
Targets	-0.096	0.090	-0.214	-0.199	-0.060
Standards	-0.100	0.170	-0.111	-0.247	-0.158
**Peak Latency**					
Targets	-0.104	-0.066	0.069	-0.236	-0.238
Standards	-0.029	-0.039	-0.121	-0.136	0.049


*^∗^ denotes statistical significance at the 0.05 level (two-tailed); ^∗∗^ denotes statistical significance at the 0.01 level (two-tailed).*

### Flanker Task

Means and standard deviations of the flanker performance and ERP indices are presented in **Table 3**. Flanker performance was skewed in favor of high performance, a pattern expected in typically functioning adults. An inclusion criterion of 50% accuracy was used for flanker performance, and no participants were excluded for low performance.

**Table 3:** Behavioral performance indices and ERP peak indices forth PZ electrode in the modified flanker task.

	Congruent trials	Incongruent trials
Response accuracy (% correct)	97.8 (3.4)	92.7 (5.1)
Reaction time (ms)	406.3 (45.2)	476.8 (43.1)
Inverse efficiency	4.2 (0.5)	4.9 (0.5)
Coefficient of variation (CV)	0.17 (0.04)	0.18 (0.04)
Peak amplitude (μV)	10.2 (3.4)	10.7 (3.7)
Peak latency (ms)	394.1 (52.0)	460.5 (55.3)


*Means are presented with standard deviations in parentheses.*

The results of the bivariate correlation analysis are included in **Table 4**. The correlation analysis indicated a number of cognitive performance variables associated with age including reaction times for congruent and incongruent trial types (*r* congruent = 0.271, *p* = 0.036; *r* incongruent = 0.290, *p* = 0.025), inverse efficiency for congruent and incongruent trial types (*r* congruent = 0.260, *p* = 0.045; *r* incongruent = 0.260, *p* = 0.045) and co-efficient of variation for incongruent trials (*r* = 0.450, *p* < 0.001). Thus, age was associated with less efficient cognitive processing as indicated by a number of performance indices. MPOD values, on the other hand, were inversely correlated only with CV for incongruent trials (*r* = –0.306, *p* = 0.018) suggesting that individuals with higher MPOD scores were more likely to process flanker information more efficiently. The processing benefit was seen specifically in terms of reliability of response patterns and only on trials that required selective processing to inhibit irrelevant distractor stimuli (i.e., during the incongruent trials). Sex and KBIT were associated with response accuracy for congruent trials (*r* = 0.260, *p* = 0.045; *r* = 0.277, *p* = 0.032) suggesting that males and individuals with higher IQ scores responded correctly to more congruent items. Sex was associated with CV for incongruent trials (*r* = –0.285, *p* = 0.027) and KBIT was associated with CV for both congruent (*r* = –0.277, *p* = 0.032) and incongruent trials (*r* = –0.394, *p* = 0.002).

**Table 4:** Bivariate correlations between participant demographic characteristics and the neuro-cognitive data from the flanker task.

	Age	MPOD	Sex	KBIT	Income
**Accuracy**					
Congruent	-0.040	-0.006	0.260*	0.277*	0.122
Incongruent	0.008	0.152	0.248	0.220	0.068
**Reaction Time**					
Congruent	0.271*	0.072	-0.066	-0.048	0.107
Incongruent	0.290*	0.027	-0.138	-0.054	0.112
**Inverse Efficiency**					
Congruent	0.260*	0.071	-0.142	-0.131	0.060
Incongruent	0.260*	0.034	-0.217	-0.148	0.050
**CV**					
Congruent	0.140	-0.090	0.002	-0.277*	-0.019
Incongruent	0.450**	-0.306*	-0.285*	-0.394**	0.142
**Peak Amplitude**					
Congruent	-0.148	0.151	-0.118	0.105	-0.088
Incongruent	-0.255*	0.259*	-0.001	0.115	-0.074
**Peak Latency**					
Congruent	0.300*	-0.065	-0.113	-0.200	0.100
Incongruent	0.154	-0.033	0.028	-0.046	-0.110


*^∗^ denotes statistical significance at the 0.05 level (two-tailed); ^∗∗^ denotes statistical significance at the 0.01 level (two-tailed).*

**FIGURE 2:**
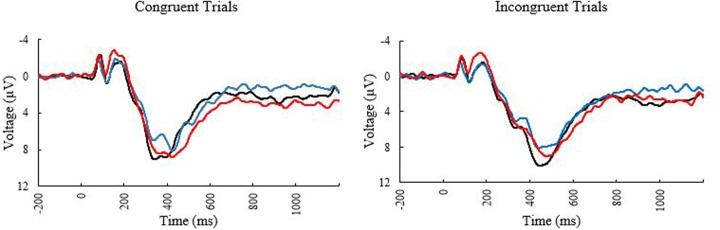
Event related potential waveforms at electrode PZ elicited during the Flanker task. Black line, younger adults; blue line, older adults with low MPOD; red line, older adults with high MPOD.

The ERP waveforms at the PZ electrode can be seen in **Figure 2**. The bivariate correlations on the ERP data revealed that age was reliably correlated with peak amplitude for incongruent trials (*r* = –0.255, *p* = 0.050) and peak latency for congruent trials (0.300, *p* = 0.020) suggesting that the older participants showed decreased amplitudes for incongruent trials, and more delayed neural responses to congruent trials. MPOD was associated with peak amplitude for incongruent trials (*r* = 0.259, *p* = 0.045), indicating that individuals with higher MPOD values showed larger amplitudes. No other demographic variables were significantly associated with the P3 data.

To further elucidate the relationships between age, MPOD, and the cognitive and P3 variables, HLRs were conducted on the two variables that were significantly associated with age and MPOD: peak amplitude and CV for incongruent trials. The results of these models are summarized in **Table 5**. When peak amplitude was used as the dependent variable and age was entered as step 1, the resulting model was statistically significant (*R*^2^ = 0.065, *F* = 4.020, *p* = 0.05), and age served as a significant predictor of peak amplitude (β = –0.255, *t* = –2.005, *p* = 0.05, CI = –0.334,0.000). However, when MPOD was added into step 2, both age (β = –0.212, *t* = –1.664, *p* = 0.102,CI = –0.307,0.028) and MPOD (β = 0.218, *t* = 1.710, *p* = 0.093, CI = –0.563, 7.161) failed to significantly predict peak amplitude, though the overall model was significant (*R*^2^ Δ = 0.046, *F* = 3.539, *p* = 0.036), suggesting that the relationship between age and PZ peak amplitude is at least partially accounted for by MPOD.

**Table 5:** Summary of regression analyses for the effects of age and MPOD on neuro-cognitive flanker variables.

	Incongruent peak amplitude			Incongruent coeff of variation		
		
Step and Variable	β	Δ*R*^2^	Model *p*	β	Δ*R*^2^	Model *p*
**Step 1**		0.065	0.050		0.203	0.000
Age	-0.255	–	0.050	0.450	–	0.000
**Step 2**	–	0.046	0.036	–	0.049	0.000
Age	-0.212	–	0.102	0.406	–	0.000
MPOD	0.218	–	0.093	-0.226	–	0.058


When CV was used as the dependent variable, step 1 resulted in a statistically significant model (*R*^2^ = 0.189, *F* = 14.749, *p* = .000) and age was a significant predictor of CV (β = 0.450, *t* = 3.840, *p* = 0.000, CI = 0.001,0.005). Step 2 of the model resulted in a significant model (*R*^2^ Δ = 0.058, *F* = 9.597, *p* = 0.000). In addition, age remained a statistically significant predictor (β = 0.406, *t* = 3.476, *p* = 0.001, CI = 0.001,0.004) whereas the added effect of MPOD was only moderately significant (β = –0.226, *t* = –1.935, *p* = 0.058, CI = –0.070,0.001).

### Go-Nogo

**FIGURE 3:**
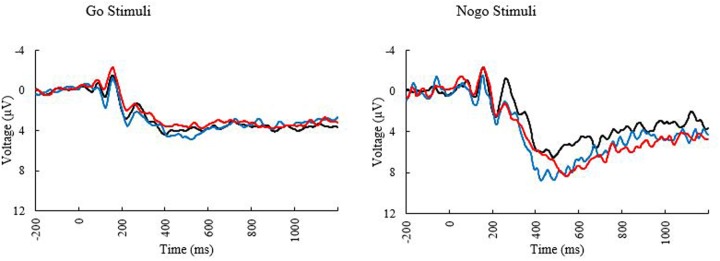
Event related potential waveforms at electrode PZ elicited during the go/no-go task. Black line, younger adults; blue line, older adults with low MPOD; red line, older adults with high MPOD.

Only a subset of participants had go-nogo data appropriate for analysis (*N* = 55, 27 females). Three participants did not complete the task or had unusable ERP data and two participants were excluded for having peak amplitudes that were outliers (Hoaglin and Iglewicz, 1987). Means and standard deviations can be seen in **Table 6**. KBIT and MPOD scores were normally distributed (Shapiro–Wilk ≥ 0.984, *p* ≥ 0.687). Age and income were not normally distributed, and the corresponding plots suggested good distribution of ages and incomes. The waveforms elicited at the PZ and FCZ electrodes can be seen in **Figure 3**. The results of the bivariate correlations (**Table 7**) revealed no significant correlations between the demographic variables of interest and the amplitudes or latencies of either the P3 or N2 components of the ERP waveform. (all *p*’s ≥ 0.095).

**Table 6:** Behavioral performance and ERP peak indices in the go-nogo task.

	Go/NoGo Task	
	
	Go stimuli	Nogo stimuli
Response accuracy (% correct)	92.2 (8.9)	65.9 (18.9)
Reaction time (ms)	415.0 (61.3)	-
Inverse efficiency	4.6 (1.0)	
Peak amplitude (μV)		
N2	-3.1 (3.6)	-4.5 (5.1)
P3	6.5 (3.5)	11.0 (5.7)
Peak latency (ms)		
N2	249.9 (31.9)	257.7 (37.7)
P3	509.1(123.4)	527.1(101.7)


*The N2 indices are reported for electrode FCZ and P3 indices are reported for the PZ electrode. Means are presented with standard deviations in parentheses.*

**Table 7:** Bivariate correlations between participant demographic characteristics, MPOD, and the neuro-cognitive data from the go-nogo task.

	Age	MPOD	Sex	KBIT	Income
**Accuracy**					
Go Stimuli	-0.046	-0.107	0.142	0.228	0.226
Nogo Stimuli	0.142	0.023	-0.137	0.024	0.113
**Reaction Time**					
Go Stimuli	0.210	0.172	-0.043	-0.036	0.114
Nogo Stimuli	-	-	-	-	-
**Inverse Efficiency**					
Go Stimuli	0.152	0.198	-0.104	-0.145	-0.061
Nogo Stimuli	-	-	-	-	-
**Peak Amplitude**					
P3					
Go Stimuli	-0.169	0.243	0.017	-0.240	-0.002
NoGo Stimuli	-0.070	0.102	0.088	-0.163	-0.035
N2					
Go Stimuli	0.067	0.059	-0.120	-0.227	-0.127
NoGo Stimuli	0.095	0.177	-0.137	-0.189	-0.002
**Peak Latency**					
P3					
Go Stimuli	-0.057	0.016	0.104	-0.185	-0.192
Nogo Stimuli	-0.130	0.167	0.192	-0.001	-0.220
N2					
Go Stimuli	-0.003	0.054	-0.002	-0.104	-0.040
Nogo Stimuli	-0.186	-0.089	0.074	-0.079	-0.040


*^∗^ denotes statistical significance at the 0.05 level (two-tailed); ^∗∗^ denotes statistical significance at the 0.01 level (two-tailed).*

## Discussion

The aim of the present study was to investigate the relationships between participant age, retinal carotenoids, and inhibition-related components of the ERP waveform throughout early and middle adulthood. Our findings are consistent with our hypotheses that age and retinal carotenoid accumulation are differentially related to performance and neurolectric indices of attentional control. However, the benefits of greater retinal carotenoids appeared to be selective for intraindividual variability and attentional resource allocation during the flanker task, rather than the in the oddball or the go/nogo tasks. Our data revealed that there were general performance benefits of age and MPOD, and that these effects were present by middle adulthood. Younger participants were more likely to perform the flanker task more quickly, more efficiently, and more consistently than older participants. MPOD scores were also associated with better performance, but the benefit of MPOD appeared more selective than that of age. Participants with higher MPOD scores were more likely to perform the flanker task with a consistent speed, although only in incongruent flanker trials, suggesting that the effect may be specific to conditions in which attentional inhibition demands are high. Secondly, participants’ P3 indices for the flanker task were correlated with age and MPOD. Younger adults were more likely to show larger P3 amplitudes, as were participants with higher MPOD scores, suggesting that those who were younger and had higher levels of retinal carotenoids were able to dedicate greater cognitive resources in the task. These results were selective to incongruent trials, where attentional inhibition is employed and were not shown in the go-nogo task indexing response inhibition.

These findings are consistent with previous literature in cognitive aging and health. A substantial body of work demonstrates that older adults show a smaller P3 peak amplitude compared to younger adults. This has been shown in a variety of paradigms and across sensory modalities (Smith et al., 1980; Pfefferbaum and Ford, 1988; Kugler et al., 1993; Morgan et al., 1999; Murphy et al., 2000). Furthermore, several studies have examined the P3 across adulthood and suggest that P3 aging effects may begin before the onset of late adulthood (Picton et al., 1984; Mullis et al., 1985; Iragui et al., 1993; Polich, 1996; Mott et al., 2014), as our data would suggest. It is possible that the smaller P3 amplitudes shown in older adults are the result of a lack of compensatory mechanisms employed that emerge as early as middle adulthood. Prior work comparing young and old high and low cognitive performers showed the increased amplitude in older high performers, but lower amplitudes in low performers which suggests that ERP amplitude may be associated with neural compensatory mechanisms that appeared lacking in our sample (Riis et al., 2008). This view is consistent with some explanations of fMRI work showing increased activity across brain regions in elderly samples (Cabeza, 2002). There has also been substantial literature to indicate that latency effects are present in cognitive aging (Pfefferbaum et al., 1980; Picton et al., 1984; Pfefferbaum and Ford, 1988; Curran et al., 2001; Walhovd and Fjell, 2003; Vallesi, 2011), effects consistent with the significant relationship between age and latency to congruent flanker stimuli in our data.

The relationship between MPOD and enhanced neuro-cognitive function is also consistent with prior literature. Several studies have established a relationship between retinal carotenoids and superior cognitive processing in older (Johnson et al., 2008, 2013; Feeney et al., 2013; Renzi et al., 2014; Vishwanathan et al., 2014a) and middle aged (Kelly et al., 2008) adults. Our data are consistent with these findings and suggest that the age-related protective properties of L and Z may begin in early to middle adulthood. This finding is not surprising. Higher MPOD is directly related to a healthy diet (Krinsky and Johnson, 2005) and healthy eating patterns have been shown to be related to better cognition across adulthood (e.g., Holloway et al., 2011). While ours is the first study to examine the relationship between retinal carotenoids and an in-vivo, brain-based measure of neuro-cognition like ERPs, the finding that higher MPOD was related to larger P3 amplitudes is an effect consistent with prior work in health-related fields. However, our results extend the existing literature by providing novel evidence for the age-related protective effects of MPOD on attentional control in young and middle-aged adults.

The results of the regression models suggest that the roles of MPOD and age in explaining variance in neuro-cognitive performance differs depending on the domain that is examined. In the behavioral domain, where CV was used as the dependent measure, age showed a robust effect independent of the effect of MPOD. This suggests that while MPOD was a moderately associated with CV performance, its effects are independent of the impact of age. Similar aging effects have been seen in other domains of cognition, such as IQ (Ardila, 2007). In the neuroelectric domain, however, the addition of MPOD into the model weakened the influence of age on P3 amplitude, suggesting that MPOD may partially influence the relationship between age and P3 amplitude and may serve as a protective factor against typical aging effects. This result is consistent with reports suggesting that cognitive aging is mediated by sensory acuity. For example, Lindenberger and Baltes (1994) found that 49% of the variance in age-related change in IQ scores was accounted for by auditory and visual acuity. Lovden and Wahlin (2005) likewise showed that visual acuity accounted for roughly half of the variance in general cognitive ability across adulthood. In this view, it is reasonable that MPOD, which has been consistently linked to visual health, (Tan et al., 2008; Junghans et al., 2001; Pintea et al., 2011; Ozawa et al., 2012; Vishwanathan and Johnson, 2013) may be an indicator of visual acuity. Indeed, higher levels of lutein and zeaxanthin have been directly linked to superior visual performance (Stringham and Hammond, 2008; Johnson, 2014; Schalch, 2014).

Our data also suggest that domains in which aging and MPOD have some combined effects on cognition are relatively selective. In our cognitive performance indices, age and MPOD were associated independently with very few measures of the oddball and none of the measures of the go-nogo task. This is perhaps unsurprising, since one might expect age effects in trials in which inhibition demands are high, such as those in the go-nogo task. However, because participants are asked to inhibit a behavioral response (i.e., pressing a button to a rare target), behavioral data are not collected on the nogo stimuli that require the highest levels of inhibitory control. Thus, the go-nogo task may not have sufficiently sensitive performance indices. However, one may still expect to see aging effects in regard to the N2 and P3, since the neural signature is present without a motor response. This effect was not shown in our data. Thus, our data suggest that the protective role of MPOD against aging-related effects may be selective for attentional inhibition, rather than for response inhibition or general selective attention as indexed by the oddball task. Prior behavioral and ERP work has shown that various forms of cognitive inhibition may be affected differentially and independently in aging, (Andrés et al., 2008; Anguera and Gazzaley, 2012), although none of these studies have used the flanker task as an index of inhibition. Furthermore, no studies to date have examined different forms of cognitive inhibition in relationship to retinal carotenoids. Thus, it is unclear whether the specificity seen in our data is a result of task demands, a result of MPOD selectively affecting attentional inhibition over selective attention or response inhibition, or a result of attentional inhibition being impacted by cognitive decline at an earlier point in the course of adulthood than the other attentional constructs measured.

There are several limitations to the present study and future work could benefit from expanding on the work in a number of ways. The present is study is cross-sectional in design and therefore is not suitable for directly answering questions regarding causality. While we attempted to elucidate on the role of age and MPOD using statistical measures (i.e., HLR), to determine true causal mechanisms it is necessary to implement interventions involving L and Z rich diets or supplementation. Furthermore, our study was limited in the age range that was utilized. While aging effects were seen even in our sample of early to middle aged adults, a more conclusive picture would emerge from examining participants across the lifespan in both cross-sectional and longitudinal designs. Longitudinal studies would also serve to answer important questions about the developmental trajectory of nutritional effects on neuro-cognitive function. Finally, there is a need to examine more exhaustive cognitive domains and paradigms in order to ascertain the reliability of the selectivity of the associations that were shown our data.

## Conclusion

Through our study, we sought to explore the relationships between aging, MPOD, and the cognitive and underlying neuro-electric indices in three attentional tasks. Our results showed robust age affects in the attentional inhibition task, but no relationships between aging and our response inhibition task. Furthermore, MPOD appeared to influence the relationship between aging and P3 amplitude in the attentional inhibition task suggesting that diet may serve a protective role in typical aging effects on inhibition. The results reported herein have important implications for the association between neuro-cognitive health and retinal carotenoids, and dietary quality by extension. While some age-related cognitive decline is to be expected in healthy aging, our data suggest that these effects may be less pronounced among adults with greater retinal carotenoid status, a marker of dietary patterns characterized by greater intake of green and leafy vegetables. Furthermore, these practices may provide neuro-cognitive benefit before the onset of older age, in early to middle adulthood. Future experimental clinical trials are needed to determine whether changes in retinal carotenoid status moderate the influence of age-related neurocognitive decline across the lifespan.

## Ethics Statement

The study was approved by the Institutional Review Board at the University of Illinois. Participants provided verbal and written consent.

## Author Contributions

AW analyzed the data and prepared the first draft of the manuscript. CE, NB, MC, AC, and GR implemented data collection and contributed to the manuscript development. BH and LR-H provided important contributions to the content of and assisted in revising the manuscript. NK conceptualized and supervised the design and implementation of the study and manuscript. All authors contributed to, and accept responsibility for, the research described in this manuscript.

## Conflict of Interest Statement

The authors declare that the research was conducted in the absence of any commercial or financial relationships that could be construed as a potential conflict of interest.
